# Additional organic and bacterium fertilizer input regulated soybean root architecture and dry matter distribution for a sustainable yield in the semi-arid Region of China

**DOI:** 10.1371/journal.pone.0305836

**Published:** 2024-07-17

**Authors:** Yu Liu, Chuhua Liu, Lichao Wei, Xudong Zhang, Qinhui Liu, Jiling Bai, Xiaolin Wang, Suiqi Zhang

**Affiliations:** 1 College of Life Sciences, Yulin University, Yulin, China; 2 Engineering and Technology Research Center of Water Saving for Crops in Arid Area of Northern Shaanxi, Yulin, China; 3 State Key Laboratory of Soil Erosion and Dry Land Farming on the Loess Plateau, Northwest A&F University, Yangling, Shaanxi, China; St. Pius X College, Rajapuram, Kasaragod-Kannur University, INDIA

## Abstract

In the dryland area of the Loess Plateau in northwest China, long-term excessive fertilization has led to soil compaction and nutrient loss, which in turn limits crop yield and soil productivity. To address this issue, we conducted experiments using environmentally friendly organic fertilizer and bacterium fertilizer. Our goal was to investigate the effects of additional organic and bacterium fertilizer inputs on soil water migration, crop root architecture, and yield formation. We implemented six different fertilizer strategies, namely: N_m_ (mulching, N 30 kg/ha), NPK_1m_ (mulching, N 60 kg/ha; P 30 kg/ha; K 30 kg/ha), NPK_2m_ (mulching, N 90 kg/ha; P 45 kg/ha; K 30 kg/ha), NPK_Om_ (mulching, N 90 kg/ha; P 45 kg/ha; K 30 kg/ha; organic fertilizer 2 t/ha), NPK_Bm_ (mulching, N 60 kg/ha; P 30 kg/ha; K 30 kg/ha; bacterium fertilizer 10 kg/ha), and N (N 30 kg/ha; no mulching). The results revealed that the addition of bacterium fertilizer (NPK_Bm_) had a positive impact on soybean root system development. Compared with the other treatments, it significantly increased the total root length, total root surface area, and total root length density by 25.96% ~ 94.89%, -19.63% ~ 36.28%, and 9.36% ~ 28.84%, respectively. Furthermore, NPK_Bm_ enhanced soil water consumption. In 2018, water storage during the flowering and podding periods decreased by 12.63% and 19.65%, respectively, while water consumption increased by 0.97% compared to N_m_. In 2019, the flowering and harvest periods decreased by 23.49% and 11.51%, respectively, while water consumption increased by 0.65%. Ultimately, NPK_Bm_ achieved high grain yield and significantly increased water use efficiency (WUE), surpassing other treatments by 76.79% ~ 78.97% and 71.22% ~ 73.76%, respectively. Subsequently, NPK_1m_ also exhibited significant increases in yield and WUE, with improvements of 35.58% ~ 39.27% and 35.26% ~ 38.16%, respectively. The use of bacterium fertilizer has a profound impact on soybean root architecture, leading to stable and sustainable grain yield production.

## Introduction

Soil microorganisms play a crucial role in the growth and development of dryland crops. They are an essential component of leaves during plant growth [1, 2], and changes in available nutrients in the soil microbial environment significantly affect the physiological adaptability of plants to abiotic stress [3]. The storage and cyclic utilization of soil microorganisms have a decisive impact on crop nutrient absorption, the migration of rhizosphere microbial ions, and the stability of productivity in cultivated soil [4]. Overall, soil microorganisms effectively stimulate hormone synthesis and promote the growth of plant leaves and roots, thereby enhancing the conversion efficiency of photosynthetic products for sustainable production [5].

In the loess dryland area, soil erosion is a serious issue, resulting in low soil quality [6, 7]. Excessive input of nitrogen fertilizer aims to improve agricultural productivity but leads to nitrogen redundancy and loss of available nitrogen into deeper soil layers [8]. Green efficient fertilization can optimize the physical composition and environmental quality of the soil, alleviate organic matter degradation, and further enhance the fertility and productivity of low-yield fields [9, 10]. Previous studies have shown that bacterium fertilizer consists of functional microorganisms, microbial bacteria, and organic matter [11]. Adding an appropriate amount of bacterium fertilizer to reduce the use of nitrogen fertilizer can help reduce soil pollution caused by excessive nitrogen and promote the increase of soil microorganisms. It also helps maintain a dynamic balance in the soil microorganism quality library and has a positive effect on the accumulation of plant organic carbon [12]. The root system plays a crucial role in the interaction between plants, soil, microorganisms, and the environment [13]. Plant roots secrete various metabolites, providing carbon sources that stimulate microbial reproduction [14]. When microbial fertilizer was added to Miscanthus instead of 30% nitrogen fertilizer, a significant enrichment of bacteria was observed in the root system, which greatly improved microbial activity in the rhizosphere [15]. In maize, the addition of compound microbial agents not only increased the diversity of soil microbial communities but also improved root structure, optimized the spatial distribution of roots, and enhanced the self-transformation of maize nutrients after absorption [16]. Furthermore, adding organic manure or bacterium fertilizer to soybeans during the growth period can effectively promote nutrient absorption by plants and optimize root structure. The vigorous root system provides a guarantee for material transformation, seed biomass accumulation, and increased yield of soybeans [17]. These aspects of scientific research are crucial for reducing nitrogen intake, improving crop water and nutrient uptake, and enhancing root development through the substitution of chemical fertilizers with organic and bacterium fertilizers in field experiments. The objective of this study was to identify a new model of green fertilization that can achieve high soybean yields in northern China.

## Materials and methods

### Field experiment sites

**Fig 1:**
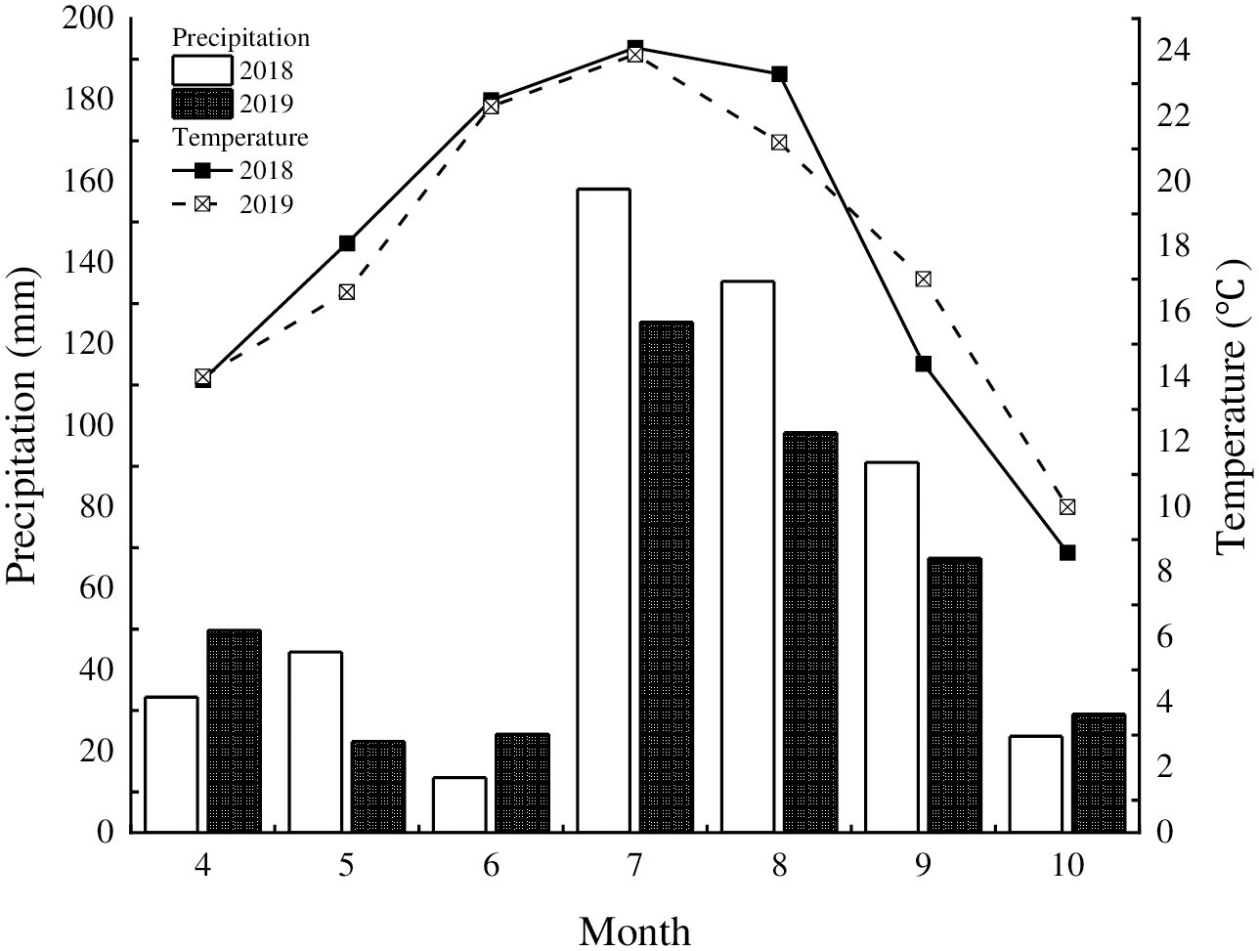
Changes in rainfall and average temperature during soybean growth period.

The experiment took place from 2018 to 2019 at Shiyaoze Village, Hengshan Town, Hengshan County, Yulin City, Shaanxi Province in northwestern China. The coordinates of the experimental site are 37°56’28.46" N, 109°21’46.81" E, with an altitude of 1232 m. This region is characterized as arid and semi-arid, and traditional agriculture is the primary mode of production. The soil type is loessial soil silty sand loam, which is uniform and loose in structure. The soil at the site has the following nutrient contents: 3.2 g/kg of organic matter, 0.3 g/kg of total nitrogen, 6.2 mg/kg of available phosphorus, and 66.0 mg/kg of available potassium. The average annual temperature at the experiment site is 8.3°C, and the mean annual precipitation is 446.8 mm. The rainfall distribution is highly uneven, with approximately 60% ~ 80% of the precipitation occurring from July to September (Fig 1).

The field study was carried out on the official land which belonged to Shiyaoze Village, Hengshan Town, Hengshan County, Yulin City, Shaanxi Province, permission was given after the research application passed verification. During the field study, none of the endangered or protected species were involved. No specific permissions were required for conducting the field study because it was not carried out in a protected area.

### Experimental design and treatments

The soybean cultivar used in the experiment was Fendou 78. The sowing of soybeans took place on May 8th, and the harvest was conducted on October 13th for four consecutive growing seasons (2018 to 2019). The experimental plots were designed to be 5 meters long and 4 meters wide. The soybeans were cultivated using wide-narrow spacing, with a row ledge of 50 cm, row spacing of 28 cm, and plastic film mulch of 60 cm. The experiments were replicated three times using a random block design. Fertilization was carried out using a combination of chemical fertilizer, organic fertilizer, and bacterium fertilizer under different treatment conditions. The treatment without film mulch and only nitrogen fertilizer application is referred to as the N, as shown in Table 1.

**Table 1:** The experiment design and fertilization amount.

Treatment	Film mulched	N (kg/ha)	P_2_O_5_ (kg/ha)	K_2_O (kg/ha)	Organic manure (t/ha)	Bacterium fertilizer (kg/ha)
N	×	30				
N_m_	○	30				
NPK_1m_	○	60	30	30		
NPK_2m_	○	90	45	30		
NPK_Om_	○	90	45	30	2	
NPK_Bm_	○	60	30	30		10

Note: × is for without film mulched and ○ is for film mulched.

### Soil water storage calculation

Soil samples were collected using a soil auger during each of the four growing seasons. The sampling was done at 20 cm intervals throughout the soil profile, ranging from 0 to 100 cm in depth. After collection, the soil samples were dried and the weight was determined using a weighing method. To calculate soil water storage, the following equation was used:

$$W=v\rho_{b}h$$ (1)

Where *w* is the soil water storage, *v* is the soil moisture content, *ρ*_*b*_ is the soil bulk density, *h* is the soil layer thickness. By calculating the soil water storage, we can assess the water availability and moisture levels in the soil profile.

### Root morphological parameters measurement

Five typical plants were selected for root sampling in the filling period. The root parameters were measured using a scanner (Epson Perfection V700, Seiko Epson Crop, Suwa, Japan) and analyzed using WinRHIZO (Regent Instrument Inc., Quebec, QC, Canada) was obtained data of the total root length (RL), root surface area (SA), root length density (RLD), the average diameter of root (ADM) and root volume (RVM).

### Dry matter accumulation measurement

Three typical plants were selected for sampling in the filling period. After manual processing in the laboratory, the samples were desiccated at 105°C for 0.5h. Then, the samples were dried to a constant weight at 80°C, and the dry weight was calculated.

### Evapotranspiration (ET) and Water Use Efficiency (WUE) calculation

Soybean plants of 3m^2^ were selected from each plot for yield determination in the maturation stage. The ET and WUE were calculated using the field soil water balance equation:

ET=ΔS+P (2)

WUE=Y/ET (3)

Where WUE is the water use efficiency of the crop, Y is the crop yield, P is the precipitation, and ΔS is the difference between the soil water storage at maturity and before sowing.

### Data statistical analysis

Excel 2010 was used to analyze data and SPSS Statistics 20.0 was used to assess univariate analysis (ANOVA). The least significant difference test was set at p ≤ 0.05 in SPSS. The figures were created using Origin Pro 9.2.

## Results

### Soil water storage

**Fig 2:**
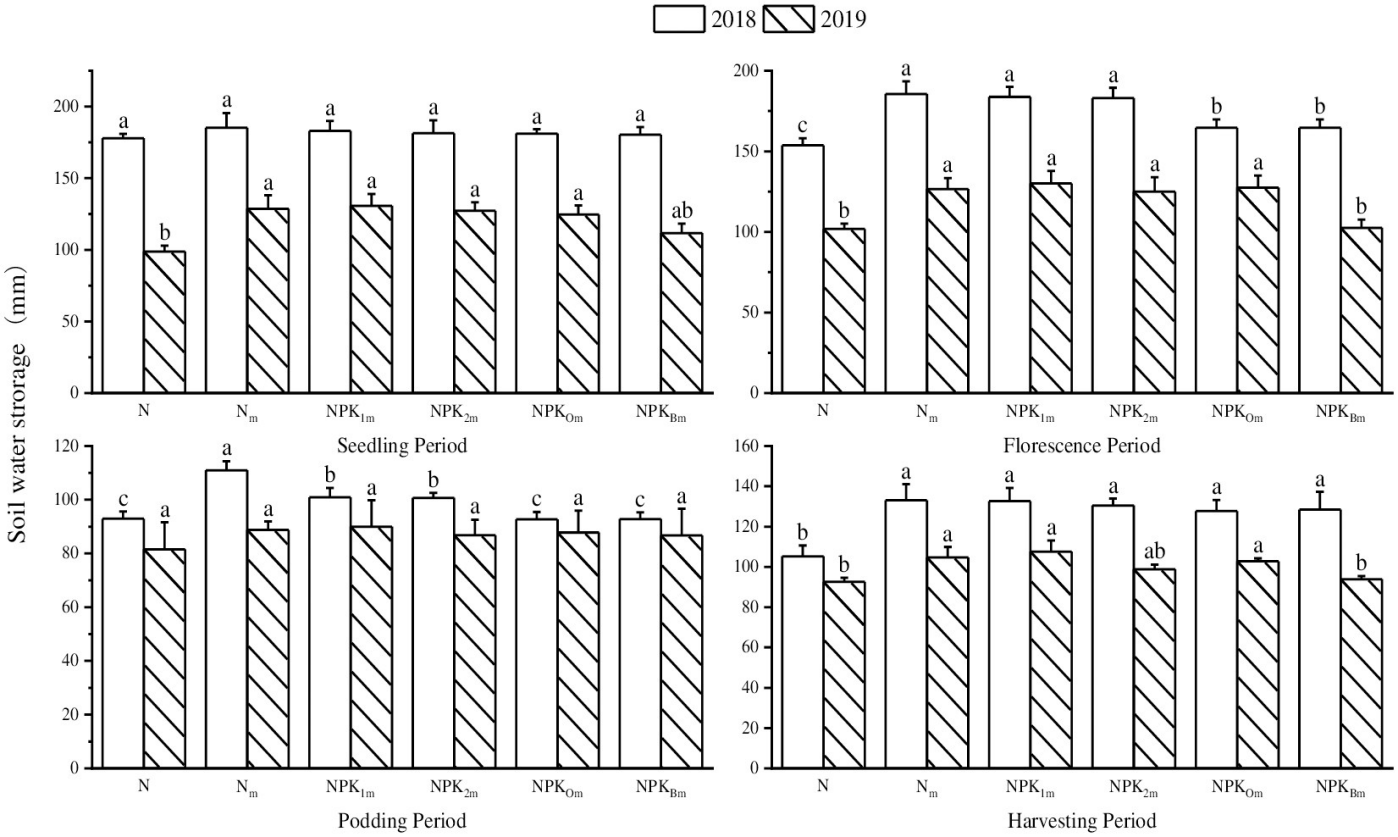
Effects of different fertilization treatments on soil water storage in 0 ~100 cm of soil depth. The values followed by different letters (a, b, and c) indicate significant differences in the same experimental year among conditions at p < 0.05, n = 3, the same below.

In 2019, the soil water storage of N_m_, NPK_1m_, NPK_2m_, NPK_Om_ and NPK_Bm_ at the seedling stage were significantly increased by 13.16% ~ 32.39% compared with N (Fig 2). The soil water storage of N_m_, NPK_1m_, NPK_2m_ and NPK_Om_ increased significantly in the florescence period. On the contrary, the soil water storage decreased in NPK_Bm_, which was conducted with additional bacterium fertilizer input. In the 2018 podding period, the water consumption of soybean pod growth decreased, resulted in an increase of rainwater infiltration and soil water storage, especially in NPK_Bm_, NPK_Om_ and N, and significantly reduced by 11.99% ~ 16.43% compared with N_m_. The total water storage decreased firstly and mostly in the podding period and then increased in the harvesting period when the root physiological activities were smoothly stopped.

### Root morphological parameters

**Fig 3:**
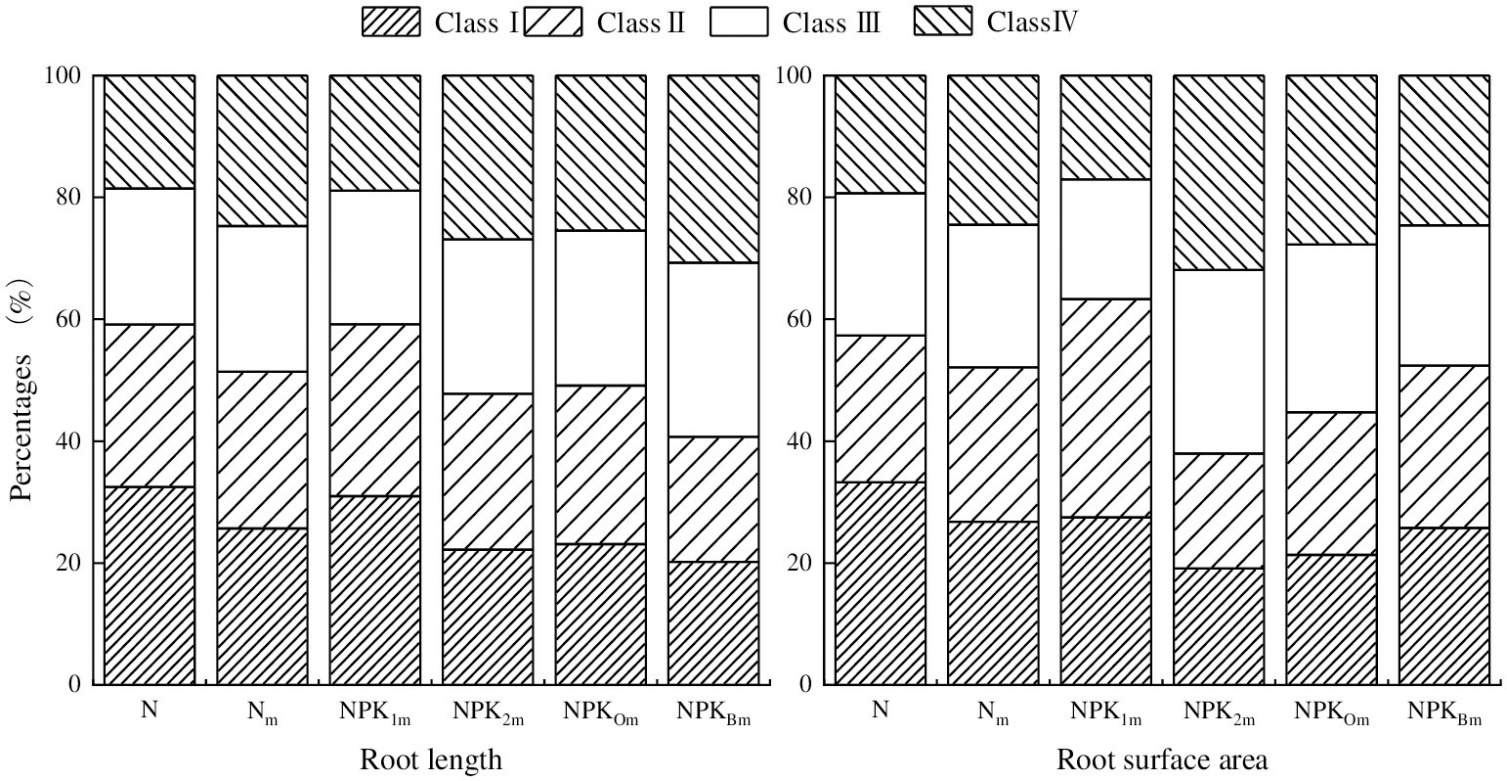
Effects of different fertilization treatments on root length (RL) and root surface area (SA) in the harvesting period. Interval levels for RL and SA were defined based on root diameter (RD, mm) size: Level I: RD 0.0–0.5mm; Level II: RD 0.5–1.0mm; Level III: RD 1.0–1.5mm; Level IV: RD > 1.5mm.

The total root length (RL) and root surface area (SA) followed a similar trend of change under the five different fertilization conditions, except for NPK_2m_ (Fig 3). In the III and IV diameter interval levels of RL, RL in NPK_Bm_ with bacterium fertilizer increased by 102.45% and 161.93% compared with N. In addition, the total RL and SA of NPK_Bm_ increased by 58. 17% and 28. 62% compared with N, respectively.

### Soil depth and Root Length Density (RLD)

**Fig 4:**
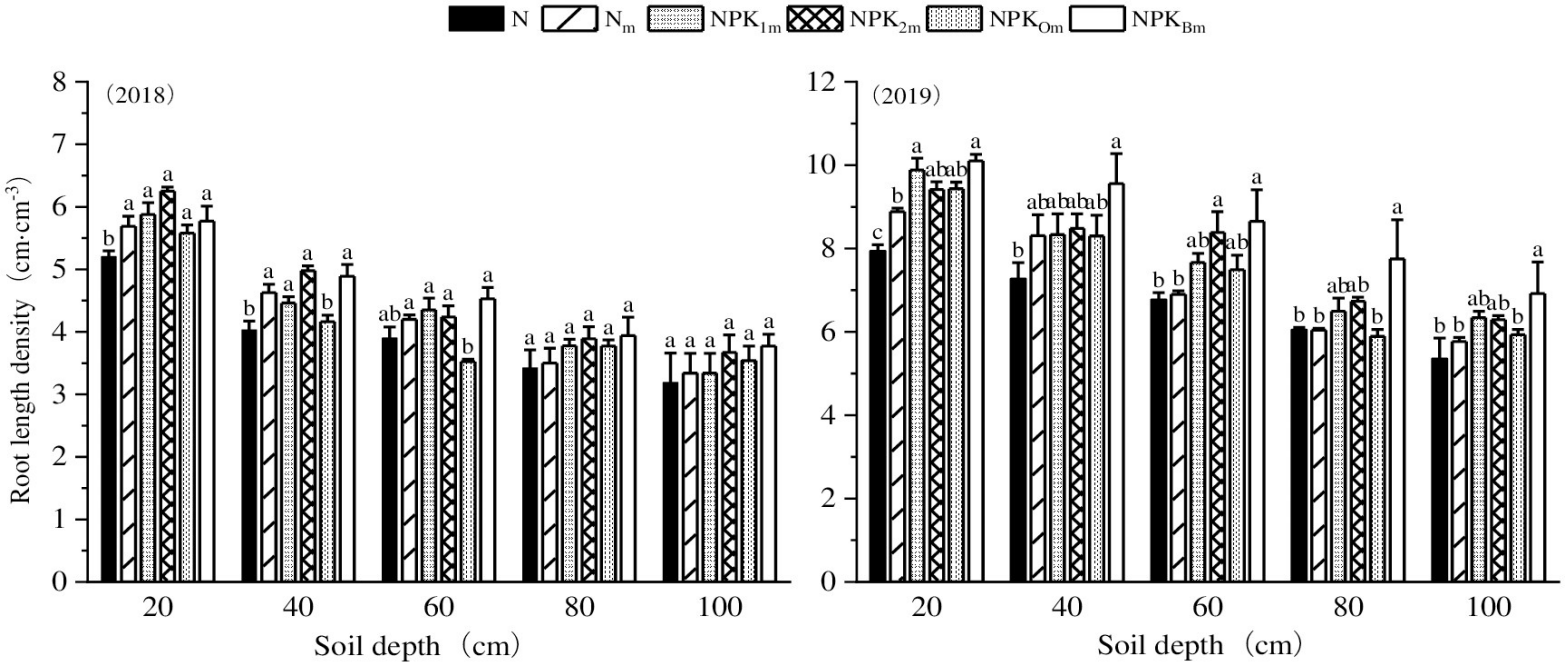
Effects of different fertilization treatments on root length density (RLD) in 0–100 cm of soil depth in the harvesting period.

In the 2018–2019 years, the root length density (RLD) in the soil depth of 0–40 cm was significantly higher than that in the 60–100 cm soil depth (Fig 4). Both nitrogen fertilizer and bacterium fertilizer had a positive and significant effect on RLD. Specifically, in the soil depth of 0–100 cm, the RLD of treatments NPK_1m_, NPK_2m_, and NPK_Bm_ showed a significant increase of 102.11% ~ 128.84% compared to N, N_m_, and NPK_Om_. In the 2018 year, the RLD of N_m_, NPK_1m_, NPK_2m_, and NPK_Bm_ at the soil depth of 0–60 cm increased by 10.59% to 17.82% compared to treatment N. Additionally, in 2019, the RLD showed a significant increase compared to that in 2018.

### Correlation analysis of dry matter accumulation and root parameters

**Fig 5:**
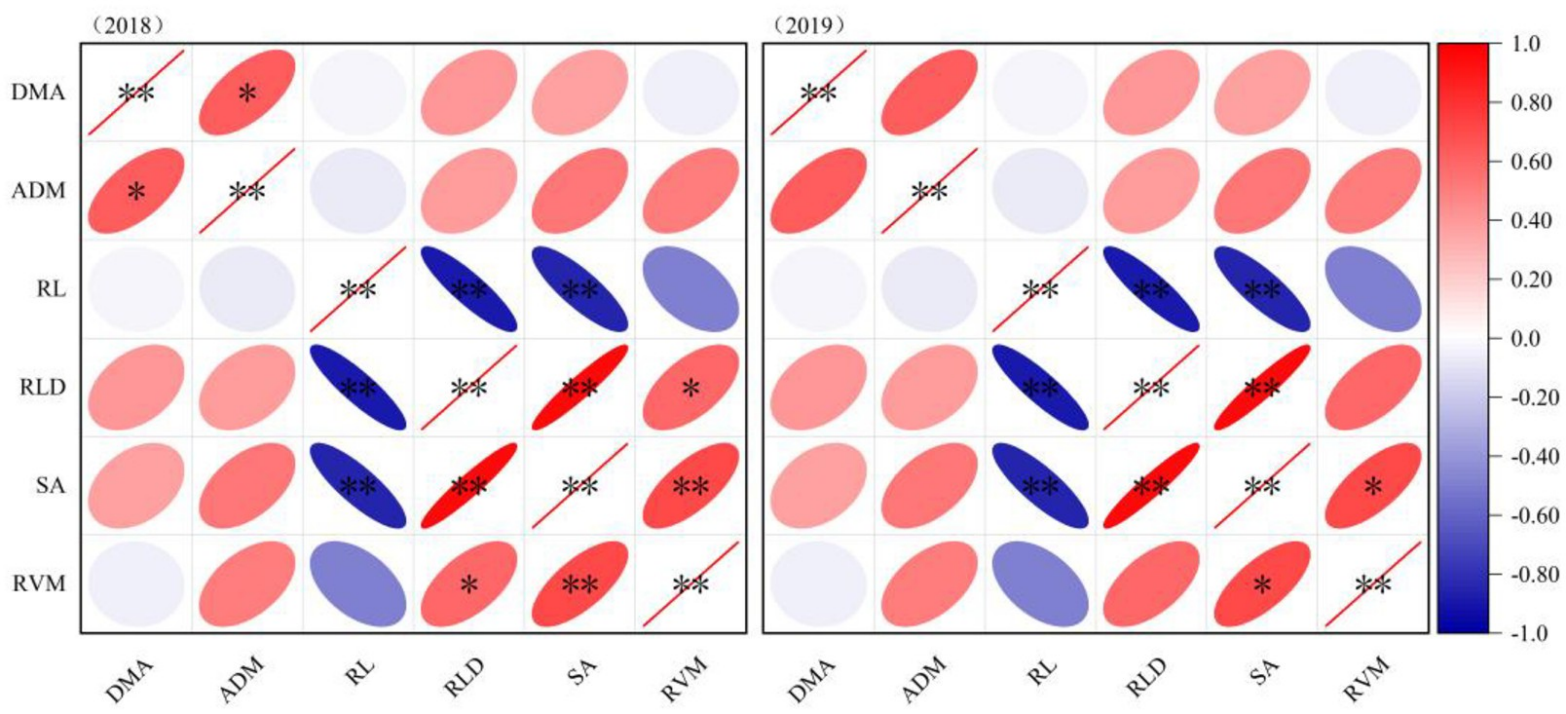
Correlation analysis between soybean dry matter accumulation and roots system in the harvesting period. *Significant difference at p < 0.05; **Significant difference at p < 0.01; DMA, dry matter accumulation; ADM, average diameter of root; RL, root length; RLD, root length density; SA, root surface area; RVM, Root volume.

The correlation between DMA and ADM, RLD and RVM were observed in 2018 (Fig 5). A conclusion was drawn from the correlation analysis, that is RLD and SA always maintained a highly significant negative correlation with RL over two years (p < 0.01). On the other hand, there were some highly significant positive correlations among RLD and RVM with SA in 2018 and 2019, respectively (p < 0.05, p < 0.01).

### Yield and water use efficiency

In 2018, the yield of NPK_Bm_ was 6.62% higher compared to the yield in 2019. However, the water use efficiency (WUE) in NPK_Bm_ was 14.21% lower in 2018 compared to 2019 (Table 2). Interestingly, the WUE in NPK_Bm_, which included bacterium fertilizer, showed a significant increase over the course of two years. It increased by 81.95% and 75.88% compared to N in 2018 and 2019, respectively. Furthermore, in 2018, N_m_, NPK_1m_, and NPK_Bm_ showed a significant increase in yield of 34.70% ~78.97% compared to N. In 2019, N_m_, NPK_1m_, NPK_2m_, and NPK_Bm_ exhibited an increase in yield of 21.65% ~73.51% compared to N. Overall, it can be concluded that NPK_Bm_ has the highest potential, with the advantage of achieving high-yield. Although the yield of NPK_Bm_ decreased in 2019 compared to 2018, it still showed significant improvements in WUE compared to treatment N. This suggests that NPK_Bm_ has the potential to achieve both high yield and improved water use efficiency.

**Table 2:** Effect of different fertilization treatments on soybean yield.

Treatment	Yield (kg/ha)		ET (mm)		WUE (%)	
	2018	2019	2018	2019	2018	2019
N	2207. 34±18. 83b	2135. 54±9. 32b	468. 83a	375. 56a	4. 71c	5. 68c
N_m_	3089. 25±12. 37a	2926. 85±7. 26a	456. 27b	368. 56b	6. 77ab	7. 94ab
NPK_1m_	3074. 25±10. 49a	2854. 61±10. 80a	459. 10b	368. 33b	6. 69ab	7. 75ab
NPK_2m_	2413. 82±18. 25b	2740. 52±7. 66a	467. 91a	369. 04b	5. 35b	7. 42ab
NPK_Om_	2973. 22±17. 40ab	2252. 70±7. 86b	468. 80a	369. 02b	6. 34ab	6. 10b
NPK_Bm_	3950. 55±10. 09a	3705. 33±17. 35a	460. 70a	370. 97a	8. 57a	9. 99a

ET and WUE in the harvesting period. The data in the table are mean ± Standard error, the values followed by different letters (a, b, and c) indicate significant differences between conditions at p < 0.05, n = 5.

### Correlation analysis of yield, dry matter accumulation and root parameters

**Fig 6:**
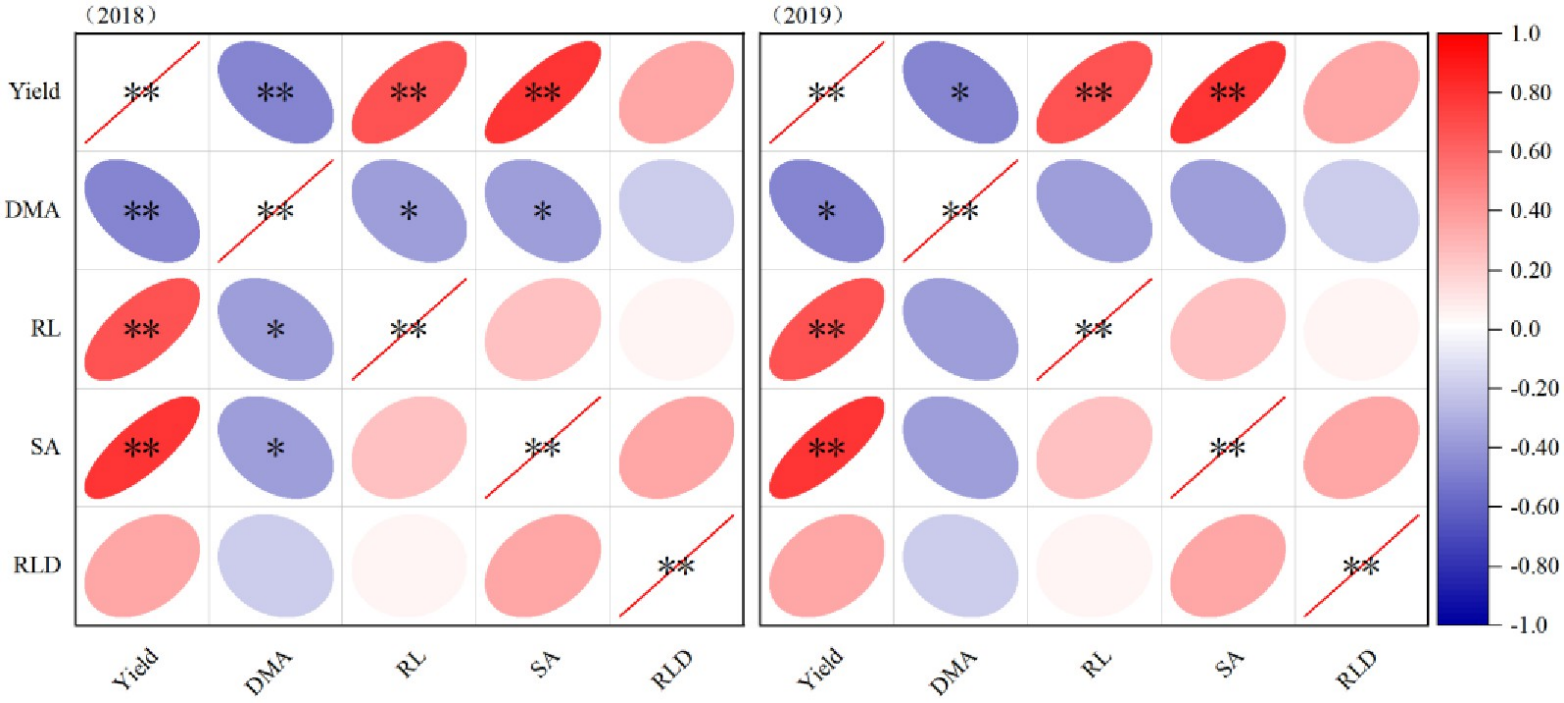
Correlation analysis among soybean yield, dry matter accumulation and roots system in the harvesting period. * Significant difference at p < 0.05; ** Significant difference at p < 0.01; DMA, dry matter accumulation; RL, root length; SA, root surface area; RLD, root length density.

A correlation analysis was conducted to examine the relationship between yield and various root parameters such as DMA (average diameter of root), RL (total root length), SA (root surface area), and RLD (root length density) over the course of two years (Fig 6). The correlation analysis revealed that RL and SA consistently maintained a highly significant positive correlation with yield throughout the two-year period (p < 0.01). This suggests that as RL and SA increase, the yield also tends to increase. Furthermore, there was a significant and highly significant negative correlation observed between DMA and yield in 2018 and 2019, respectively (p < 0.05, p < 0.01). This indicates that as yield increases, the DMA tends to decrease. These correlations provide valuable insights into the relationship between root parameters and yield. The positive correlation between RL, SA, and yield suggests that a greater root length and surface area are associated with higher crop yields. On the other hand, the negative correlation between DMA and yield indicates that high yield may have a slowed down increase on DMA. Overall, these findings highlight the importance of root parameters in influencing crop productivity.

## Discussion

The process of soil nutrient fixation and conversion to soil fertility in agroecosystems is indeed complex. Although artificial fertilization can achieve a short-term dynamic balance of the soil ecosystem, the farmland ecosystem requires a longer-term dynamic change process [19]. Through long-term location experiments in farmland ecosystems, it has been observed that a significant accumulation of available nutrients occurs during the early growth stages of crops, primarily in the cultivated soil layer. However, this accumulation also poses a risk of nutrient loss through volatilization and leaching. The addition of microbial fertilizer treatments can enhance the activity of microorganisms and increase the bioavailability of fertilizers. The root system, being a crucial vegetative organ, continues to grow deeper into the soil as the growth period progresses. This downward growth of roots helps transport nutrients to deeper soil layers. Furthermore, under the influence of microorganisms, a dynamic process of organic matter release occurs. This process promotes the absorption and utilization of nutrients by roots, creating a cycle of nutrient availability and uptake. Overall, these findings highlight the importance of considering the long-term dynamics and interactions in the soil ecosystem. By enhancing microbial activity and utilizing the root system’s nutrient transport capabilities, it is possible to improve nutrient availability, uptake, and utilization in agricultural systems [18, 19, 36]. The root system exhibits high plasticity in response to changes in the soil ecosystem and has a strong correlation with crop yield [20]. In this study, it was observed that by reducing nitrogen fertilizer application and instead using bacterium fertilizer, the growth of root length (RL) was accelerated. Specifically, the root length density (RLD) and RL of the NPK_Bm_ showed significant increases in the III ~ IV diameter interval levels. Furthermore, the NPK_2m_ had a significant impact on the total root surface area (SA) in the I ~ IV diameter interval levels. This effect may be attributed to the increased application of fertilizer, which promoted the lateral growth of soybean roots [21]. It is important to note that the architecture of the root system not only determines the plants ability to acquire water and nutrients from the soil but also influences the physiological functions of the root system [22]. The structure and distribution of the root system play a crucial role in maximizing the plant’s potential to absorb and utilize resources from its environment. Indeed, the ecological function of plants and the relationship between the root system and aboveground parts require comprehensive and in-depth long-term exploration. In this study, a significant correlation was found between dry matter accumulation (DMA) and the average diameter of roots (ADM) (p < 0.05). This correlation indicates that the biomass accumulation of the plant is influenced by the diameter of the roots. As the plant is a biological entity, the conversion of yield is regulated by the accumulation of biomass. Therefore, it is important to consider the correlations among the root system, biomass, and final yield. Exploring these correlations can provide valuable information on the mechanisms that drive plant growth and productivity. It can help identify the factors that influence biomass accumulation, such as root system architecture, nutrient uptake, and resource allocation. By understanding the relationship between the root system, biomass, and final yield, we can gain insights into optimizing crop management practices and enhancing overall plant performance [23, 24]. In this study, it was observed that there was no synchronous positive increasing trend between dry matter accumulation (DMA) and final yield. In fact, a significant negative correlation was found between DMA and yield (p < 0.05). However, there was a significant positive correlation between yield and root length (RL) and root surface area (SA) (p < 0.01). The negative correlation between DMA and yield could be attributed to increased frequency inconsistency during the harvest period [22–24]. This inconsistency may have led to variations in the timing and extent of biomass accumulation, resulting in a negative relationship with final yield. Interestingly, the correlation between biomass and root indexes (such as RL and SA) was not significant in this study. The specific reasons for this lack of correlation require further investigation. Overall, the findings underscore the need for comprehensive and in-depth long-term exploration of the plant’s ecological function and the complex relationship between the root system and aboveground parts. Understanding these relationships can provide insights into the factors influencing biomass accumulation, yield formation, and the interplay between the root system and aboveground components. Further research is necessary to gain a deeper understanding of these complex interactions.

Crops grown in the loess dryland area often face challenges such as low and unsustainable yields and high water consumption due to abiotic stresses [6, 7, 18]. Therefore, it is a great significance to explore water dynamics and transformations in the soil ecosystem under the bacterium fertilizer mode, which promotes resilient root growth and high-quality yield formation [25]. The application of bacterium fertilizers can have positive effects on the soil, such as increasing soil volume and total porosity, which can further enhance the water consumption by plants [26, 27]. In this study, it was observed that the evapotranspiration (ET) of the NPK_Bm_ was significantly increased by 1.96% ~ 2.75% compared to the other treatments over the course of two years. This increase in ET indicates a greater water consumption by the soybean plants treated with bacterium fertilizers. The study of water use efficiency (WUE) under a scientifically optimized fertilization mode can help characterize how soil hydraulic characteristics respond to the relationship between crop water transport and yield formation [28, 29]. The differences in natural rainfall, as well as the dynamic balance between upward evapotranspiration and downward water infiltration, are the main factors that influence the water balance in the soil environment. These factors, in turn, affect the WUE of soybeans [30]. Absolutely, the application of bacterium manure can indeed facilitate the coordination of the water cycle in the soybean root system. Through microbial activities and transformations, various mineral particles, inorganic substances, and organic substances in soil aggregates combine and form compounds that enter the soil. This process improves soil fertility and enhances the ability of crop roots to absorb nutrients [27, 31]. Having good soil resources is a crucial factor in the absorption and utilization of nutrients by crop roots. The nutrients acquired by the roots from the soil are then transported to the aboveground vegetative organs, ultimately promoting the efficient accumulation of carbohydrates in crops [9, 20, 24]. This nutrient transfer and carbohydrate accumulation contribute significantly to the growth, development, and productivity of the crop. In this study, it was found that the application of NPK_Bm_ with bacterium manure resulted in maximized water use efficiency (WUE). Specifically, in 2019, the WUE increased by 16.60% compared to the previous year (2018). These results indicate that the use of bacterium fertilizer enhanced the absorption of soil nutrients by soybean roots and significantly stimulated the yield potential of soybeans. Although the application of bacterium fertilizer was accompanied by high soil water consumption, it still improved the overall water use efficiency of soybean. This suggests that the increased nutrient uptake and improved yield potential outweighed the additional water requirement, resulting in a more efficient use of water resources.

Indeed, understanding the relationship between crop root system architecture, water output and transformation in the soil ecosystem, and yield is of great practical significance for achieving high-quality crop yield [32]. By comprehending these relationships, we can develop strategies to optimize crop root development and enhance water utilization efficiency, ultimately leading to improved crop productivity. The use of environmentally friendly fertilizers, such as bacterium fertilizer, can have positive effects on soil activity and the growth environment for crops. Previous studies have demonstrated that the application of bacterium fertilizer can increase the rate of water transformation at the soil-plant interface, leading to improved water availability for plants. As a result, soybean yields were significantly higher when bacterium fertilizer was used compared to treatments without bacterium fertilizer [33]. Several studies have found that the addition of bacterium fertilizer in corn-soybean rotations can have significant positive effects on plant roots and soil microbial communities. These studies have shown that the application of bacterium fertilizer enriches the soil with beneficial microorganisms, which in turn facilitates the adsorption of organic matter by plant roots. This stimulation of root system compensatory growth leads to enhanced water and nutrient uptake capabilities, ultimately resulting in increased yields for both corn and soybean crops [34, 35]. Similarly, the present study has also reached a similar conclusion regarding the application of bacterium fertilizer. It was observed that the use of bacterium fertilizer increased the abundance of microorganisms in the soil, thereby promoting interactions between soybean roots and the soil ecosystem. This enhanced the growth and resistance capabilities of soybean roots, as well as their ability to absorb nutrients from the soil. In the year 2018, adequate water availability created favorable growing conditions for crops, ensuring stable and high yields for the NPK_Bm_. However, in the following year, there was a decrease in natural rainfall, leading to a more arid environment. This arid condition stimulated the growth of crop roots, enabling them to absorb nutrients and water from deeper soil layers [36, 37]. As a result, the water use efficiency (WUE) increased, and the yield of NPK_Bm_ was maintained, with only a 4.43% reduction compared to the previous year. On the other hand, the NPK_2m_ had a low yield in 2018. This could be attributed to the possibility of excess moisture causing leaching and decomposition of the fertilizer, resulting in poor fertilizer utilization and reduced yield [37, 38]. Furthermore, the NPK_Om_ did not show a significant increase in yield. This could be due to the negative effects of organic fertilizer application, which can alter soil pH and other soil properties, thereby impacting crop growth [16, 39, 40]. Another possibility is that the microbial community in the soil absorbed a significant amount of organic matter, leading to lower available nutrients for the crops and consequently resulting in a lower yield [27, 41, 42]. Indeed, further investigation is necessary to fully understand the reasons behind the observed effects. However, it can be concluded that adding an appropriate amount of bacterium fertilizer, while reducing the use of nitrogen fertilizer, can contribute to plant growth and achieve the goal of high crop yields. The NPK_Bm_ which utilized bacterium fertilizer, exhibited better performance across various indices. Microorganisms present in the soil play a beneficial role in regulating various factors and improving the conversion rate of carbon compounds in soybeans. Moving forward, it would be valuable to conduct more detailed studies on the application rate of bacterium fertilizer. This would help develop strategies to achieve high-quality crop yields while conserving bacterium fertilizer usage and maximizing the benefits for crop growth.

## Conclusions

The root system of plants is the first organ to perceive changes in the soil environment. By applying fertilizer to regulate the soil environment and promote root morphology, the ability of soybean roots to acquire nutrients is fully stimulated. This leads to improved material conversion efficiency between the root system and the above-ground parts of the plant, ultimately resulting in increased water use efficiency (WUE) and yield. The NPK_Bm_ had the most significant effect on soybean root morphological parameters, with the total root length (RL) and surface area (SA) increasing by 58.17% and 28.62% compared to the N, respectively. Additionally, the root density of NPK_Bm_ at depths of 0–100 cm increased by 10.96% ~ 21.34% in 2018 and 27.36% ~ 31.50% in 2019 compared to the N. These positive effects on soybean roots also translated into high yields and improved efficiency. In 2018 and 2019, soybean yields and WUE of NPK_Bm_ increased by 27.88% ~78.97% and 26.59% ~ 81.95%, respectively, compared to other treatments. However, the vigorous root system also resulted in a decrease in soil water storage and an increase in crop water consumption. The water storage of NPK_Bm_ decreased by 2.75% ~ 19.65% and 2.39% ~ 23.49%, while water consumption increased by 0.97% and 0.65% compared to the N_m_ from 2018 to 2019, respectively. These findings provide a theoretical basis for understanding the interaction between fertilizer application, soybean root morphology, soil moisture, and yield in loess dryland. They also serve as a reference for studying the relationship between fertilizer application and root growth for other crops, and how it can contribute to increased yield.

## Supporting information

S1 Data The caption of minimal data set: Based on the figures and tables, all the analyzed and related data providing in the files. (XLSX)
